# EFFECT OF EMDR THERAPY ON POST-TRAUMATIC STRESS SYMPTOMS, SYMPTOM SEVERITY AND ANXIETY LEVEL IN PSYCHOTIC PATIENTS WITH AT LEAST ONE TRAUMATIC EVENTS

**DOI:** 10.1192/j.eurpsy.2023.2234

**Published:** 2023-07-19

**Authors:** E. Yıldız, Z. Z. Yıldız, M. Karadağ

**Affiliations:** 1 Psychiatrist, Private Clinic; 2 Child Psychiatrist, Gaziantep University Faculty of Medicine, Gaziantep, Türkiye

## Abstract

**Introduction:**

Eye Movement Desensitization and Reprocessing (EMDR) is a powerful psychotherapy approach developed by Francine Shapiro in 1987 when she realized that rhythmic eye movements reduce disturbing thoughts. The effectiveness of the therapy has been proven by many studies after its discovery. EMDR, which was initially used only in the treatment of Post Traumatic Stress Disorder, has become a treatment option used in the treatment of psychiatric disorders of more than 2 million people today. EMDR, which is used today, contains elements from many therapy schools andconsists of a phased protocol (Shapiro, 2018). Increasing evidence acknowledging the relationship between trauma and psychosis indicates that EMDR can be a vital addition to the treatment of psychosis (Sin & Spain, 2017; Valiente-Gomez et al., 2017). However, the effect of EMDR on psychosis has not yet been sufficiently clarified. There is alsoIt has not yet been clarified whether the curative effect on the psychotic symptoms or on the anxiety symptoms.For this reason, in our study, the effect of EMDR on these symptoms will be investigated by comparing the case and control groups.

**Objectives:**

This study aims to evaluate the effect of EMDR therapy on post-traumatic stress symptoms, schizophrenia symptom severity and anxiety level in psychotic patients with at least one traumatic event.

**Methods:**

This study is a randomized controlled, prospective follow-up study aiming to evaluate the effect of EMDR therapy on post-traumatic stress symptoms, schizophrenia symptom severity and anxiety level in psychotic patients with at least one traumatic event.Written informed consent to participate in the intervention study will be requested from all patients who meet the inclusion criteria. Consent participants receive pre-treatment (T0) measurements. After T0, participants will be randomized to EMDR or waiting list Participants will be randomly assigned to 26 people in each group. These groups will be made by the independent randomization bureau of the Parnassia Institute of Psychiatry using the scientific randomization program on the Internet (www.randomizer.org).

**Results:**

The effects of EMDR therapy on posttraumatic stress symptoms, schizophrenia symptom severity and anxiety level in psychotic patients were obtained from pretest and posttest measurements.

**Conclusions:**

More studies are needed on the effectiveness of Eye Movement Desensitization and Reprocessing (EMDR) therapy.

**Disclosure of Interest:**

None Declared

